# Screening of Aqueous Extract of *Persea americana* Seeds for Alpha-Glucosidase Inhibitors

**DOI:** 10.1155/2022/3492203

**Published:** 2022-05-14

**Authors:** Tajudeen Afolayan Lawal

**Affiliations:** Department of Biochemistry and Forensic Science, Faculty of Science, Nigeria Police Academy, Wudil, Kano, Nigeria

## Abstract

Activity of *α*-glucosidase enzyme in the gastrointestinal tract has been implicated in postprandial hyperglycaemia. If not properly controlled, postprandial hyperglycaemia might progress to diabetes mellitus, a metabolic syndrome. Diabetes is associated with many complications such as retinopathy, heart attack, nephropathy, neuropathy, stroke, and lower limb amputation. Antidiabetic medications presently in use have little effect on postprandial glycaemic excursion and hence do not bring down the blood glucose level to baseline. This study extracted, fractionated, and screened the aqueous extract of *Persea americana* seeds for hypoglycaemic potential. Inhibitory effects of the fractions and subfractions of the extract on *α*-glucosidase activity were investigated. The most active subfraction was subjected to Fourier transform infrared (FTIR) and gas chromatography mass spectroscopy (GC-MS) analysis to elucidate the active components. The active subfraction showed a significant inhibition (*p* < 0.05) on *α*-glucosidase. The subfraction competitively inhibits *α*-glucosidase (with IC50 = 09.48 ± 0.58 *μ*g/mL), though less potent than the standard drug, acarbose (IC50 = 06.45 ± 0.47 *μ*g/mL). FTIR analysis of the subfraction showed the presence of carbonyl group, hydroxy group, carboxyl group, double bonds, methylene, and methyl groups. GC-MS analysis suggests the presence of cis-11,14-eicosadienoic acid, catechin, and chlorogenic acid as the active components. In conclusion, the components obtained from this study can be synthesised in the laboratory to further confirm their hypoglycaemic activity. The most active subfraction can be explored further to confirm its inhibitory activity against the enzyme and to determine its extent in the treatment of diabetes mellitus in vivo.

## 1. Introduction

Diabetes mellitus is an endocrine metabolic disorder characterized by chronic hyperglycaemia (high blood sugar) giving rise to the risk of microvascular (retinopathy, nephropathy, and neuropathy) and macrovascular (ischaemic heart disease, stroke, and peripheral vascular disease) damage [1], with associated reduced life expectancy and diminished quality of life [2]. Inherited or acquired deficient production of insulin or ineffectiveness of the insulin produced by the pancreas causes diabetes mellitus [3, 4]. Estimation by the WHO shows that about 30 million people suffered from diabetes in 1985, the figure raised to more than 171 million in 2000, and it is predicted that the number would rise to over 366 million by 2030 [5]. The substantial increase would be in developing countries, among people aged between 45 and 64 years [6, 7]. In a World Health Organisation multinational research, 50% of people with diabetes died of cardiovascular disease, mainly heart disease and stroke [8].

Despite the fact that diabetes mellitus is spreading swiftly, the existing accessible drugs to manage the dreaded syndrome are associated with many serious side effects such as liver disorders, flatulence, abdominal pain, renal tumour, abdominal fullness, and diarrhoea [9–12].

Activity of *α*-glucosidase enzyme has been implicated in postprandial hyperglycaemia, the control of which might be helpful in regulating blood glucose levels. Alpha-glucosidase (EC 3.2.1.20) catalyses the hydrolysis of terminal, nonreducing *α*-1,4-glycosidic linkage of glucose residues from oligosaccharides and disaccharides yielding glucose units as products [13]. Alpha-glucosidase is among a number of diabetic targets, which is regarded as an important therapeutic target to manage hyperglycaemic conditions [14, 15]. The inhibitors of *α*-glucosidase enzyme actually slow down the digestion and absorption of carbohydrates and thus decrease the insulin demand [15] . In this way, postprandial hyperglycaemia could be controlled independently of insulin [16]. Currently, effective *α*-glucosidase inhibitors with low or no toxicity are highly required. Several medicinal plants have been evaluated for their potential role on *α*-glucosidase enzymes. This study screened fractions and subfractions of aqueous extract of *Persea americana* seeds (AEPAS) for *α*-glucosidase inhibitors.

*Persea americana*, which is commonly called avocado pear, is one of the medicinal plants that have been broadly used in traditional folk medicine [17]. It belongs to the family of plants called Lauraceae. In Nigeria, the plant has various local names such as “apoka,” or “ewe pia” in Yoruba; “ube-beke” or “akwukwo ube” in Igbo; and “fiya” in Hausa [18, 19]. It has been reported that the seed of *Persea americana* has various uses in phytomedicine, such as antidiarrhoea, antidysentery, toothache alleviation, anti-intestinal parasites, skin treatment, and beautification [20]. A significant decrease (*p* < 0.001) in blood glucose was observed in alloxan-induced diabetic rats treated with the crude aqueous extract of *Persea americana* seed [21]. Crude aqueous extract of *Persea americana* seed has been reported to significantly (*p* < 0.05) inhibit *α*-amylase and *α*-glucosidase [1].

Investigations have shown that *Persea americana* seed extract contains significant quantities of phenolic compounds, which makes it to be a good source of antioxidants [22]. Phenolic-rich plant extract has been attributed to the remediation of diabetic complications and alleviation of oxidative stress [23–25].

## 2. Materials and Methods

### 2.1. Materials

#### 2.1.1. Medicinal Plants

Fresh *Persea americana* fruits were obtained locally and identified at the herbarium unit of Biological Sciences Department, Bayero University, Kano, Nigeria. The seeds were assigned reference numbers.

#### 2.1.2. Equipment

The equipment used for the study are Centrifuge (800D) from Gulfex medical and scientific, England; Spectrophotometer from Spectrum Lab and 52b New life medical instrument, England; Water Bath (OLS200) from Grant Instrument (Cambridge) LTD; Weighing Balance (Scout Pro Spo402) from Ohaus Corporation, Pine Brrok, NJ, USA; FTIR (Cary630) from Agilent Technology, Malaysia; Heater (HP139920-33) from Barnstead Thermolyne, England; Glucometer (CE0088) from Roche Diagnostic GmnH, Germany; and GC-MS from Agilent 19091S-433UI Agilent Technologies, United States.

#### 2.1.3. Chemicals and Reagents

*Saccharomyces cerevisiae α*-glucosidase and p-nitrophenyl glucopyranoside (pNPGP) were from Sigma-Aldrich, USA. Para-nitrophenol, sodium dihydrogen phosphate dihydrate, and disodium hydrogen phosphate dodecahydrate were from Hopkin and Williams Chadwell Health, Essex, England. Sodium carbonate was from BDH Chemicals Ltd, Poole, England, while acarbose (Glucobay) was from Bayer AG Kaiser-Wilhelm-Allee 51368 Leverkusen, Germany.

### 2.2. Methodology

#### 2.2.1. Preparation of Acarbose Solution (0.1 mg/mL)

Acarbose (Standard Drug: Glucobay 100 mg/tablet by Bayer-antidiabetic. Each Glucobay tablet contains 100 mg of acarbose). Weight of 5 tablets (100 mg/tablet) was taken, and the average weight of the tablets was determined. Equivalent weight of 5 mg acarbose was weighed and dissolved in 50 mL of phosphate buffer, pH 6.9, agitated for 30 minutes, and filtered, and the filtrate was used within a day.

#### 2.2.2. Extraction

After washing thoroughly in running water, the succulent parts of the fresh *Persea americana* fruits were chopped off, the left-over seeds were sliced into pieces, air-dried in the shade for 14 days, and milled to coarse powder using a mortar and pestle. Exactly 50 g of the powder was soaked in 500 mL of deionised water. The mixture was allowed to stand for 48 hours with intermittent shaking and thereafter filtered with a mesh cloth. The liquid mixture was then centrifuged at 8000 rpm for 10 minutes. The crude extracts were subsequently oven-dried at a temperature of 45°C to form a powdery or solid residue, stored in a closed container, labelled as “AEPAS,” and refrigerated at 4°C.

### 2.3. Fractionation

#### 2.3.1. Column Chromatography

Silica gel of mesh size 120 G was used as the stationary phase, while varying solvent combinations were used as the eluent. A slurry was formed by mixing 200 g of silica gel in 500 mL of hexane and poured down quickly and carefully into the column, and the tap was left open during packing to allow free flow of the solvent into a beaker below. At the end of the packing, the tap was closed and left to stand undisturbed for 24 hours, after which the clear solvent on top of the silica gel was allowed to drain down to the silica gel meniscus. The flow rate of the column was noted.

Ten grams (10 g) of the dried extracts were mixed thoroughly with 20 g of silica gel and then gently layered on top of the column. Elution of the column was performed with various solvent combinations of varying polarity. The following solvents systems were used in the elution process: hexane: ethyl acetate 100 : 0, 90 : 10, 80 : 20, 70 : 30, 60 : 40, 50 : 50, 40 : 60, 30 : 70, 20 : 80, 10 : 90, and 0 : 100. For each solvent combination, the elution was performed until each solvent ratio became clear. The eluted fractions were collected in an aliquot volume of 50 mL and then pooled by thin-layer chromatography.

#### 2.3.2. Thin-Layer Chromatograph (TLC)

Pooling of the column chromatography fractions was performed using analytical thin-layer chromatography (TLC). Each fraction was spotted on a precoated aluminium silica gel plate and developed in a chromatographic tank in the appropriate solvent system. With the aid of a capillary tube, a spot of the sample was applied on the plate at 1.0 cm distance from the base of the plate, and the plate was allowed to dry at room temperature and lowered in a chromatographic tank containing the solvent system saturated with the solvent vapour. The solvent was allowed to ascend the plate until the solvent front reached about ¾ of the length of the plate. The plate was removed and allowed to dry at room temperature. It was then sprayed with a freshly prepared 0.5 mL p-anisaldehyde in 50 mL glacial acetic acid and 1 mL 97% sulphuric acid and heated at 105°C for 10 seconds to visualize the bands. The colour reaction was noted, and the relative retention factor (Rf) is calculated as follows:

(1) $$\begin{array}{c} R_{f}=\frac{\text{distance travelled by compound from origin}}{\text{distance travelled by solvent from origin}}. \end{array}$$

Each pooled fraction was tested for inhibitory activity against *α*-glucosidase.

#### 2.3.3. Preparative Thin-Layer Chromatograph (PTLC)

A dried cleaned glass plate (20 × 20 cm) was placed on the plastic base plate over a plane surface. A slurry of silica gel in water in the ratio 1 : 2 (w/v) was prepared. The slurry was stirred thoroughly for 2 minutes and poured into the applicator positioned on the head glass plate. The slurry was coated over the glass plates at a thickness of 0.25 mm. The plates were left to dry at room temperature for 30 minutes. The plates were heated in an oven at 100°C for an hour to remove the moisture and to activate the adsorbent on the plate. About 2.5 cm distance from one end of the glass plate and at least an equal distance from the edges was left. The sample was applied by means of a micropipette or syringe as streak. The streak was placed at an equal distance from one end of the plate and the adsorbent was ensured not to flake off at the sample application point. The sample was allowed to dry.

#### 2.3.4. Developing Chromatogram

The developing solvent (chloroform-methanol, 1 : 1) was poured into the tank to a depth of 1.5 cm. It was allowed to stand for at least an hour on a cover plate over the top of the tank to ensure that the atmosphere within the tank becomes saturated with solvent vapour (i.e., equilibration). After equilibration, the cover plate was removed, and the thin-layer plate (sample applied) was placed vertically in the tank so that it stands in the solvent with the streaked end dipping in the solvent. The cover plate was then replaced. The separation of the compounds occurred as the solvent moved upward. The chromatogram was developed at constant temperature to avoid anomalous solvent-running effects. Once the solvent reached the top of the plate, it was removed from the tank, dried, and then proceeded for the identification of the separated compounds.

The separated compounds were viewed under UV light, and the separated components were shown up as blue, green, and black spots. The plates under UV light were examined to locate the UV absorbing or fluorescent compounds. The fluorescent areas were scrapped, eluted with a suitable solvent (methanol), air-dried, and weighed.

#### 2.3.5. Screening for *α*-Glucosidase Inhibition

Alpha-glucosidase enzyme solution was prepared just before it was used by dissolving 0.1 mg of *α*-glucosidase enzyme in 10 mL of 20 mM phosphate buffer (pH 6.9). Evaluation of *α*-glucosidase inhibition by AEPAS was performed using the methods of [14, 26, 27] with slight modifications. Accurately 100 *μ*L of AEPAS of varying concentrations—10, 20, 40, 80, and 160 *μ*g/mL—was mixed with 100 *μ*L of phosphate buffer (pH 6.9), and 100 *μ*L of 5 mM p-nitrophenyl *α*-D-glucopyranoside (pNPG) was then added. The reacting mixture was thoroughly and gently mixed and then incubated at 37°C for 10 minutes. Thereafter, 20 *μ*L *α*-glucosidase solution was added and incubated at 37°C for another 10 minutes. The blank sample contained 40 *μ*L of phosphate buffer instead of 20 *μ*L *α*-glucosidase and 20 *μ*L of AEPAS. The control sample contained 20 *μ*L of phosphate buffer in place of 20 *μ*L of AEPAS sample. After 10 minutes of incubation, 100 *μ*L Na_2_CO_3_ (200 mM) was added to each sample to end the reaction. Absorbance of each sample was then determined spectrophotometrically at 405 nm. Acarbose was used as a positive control. The experiments were run in triplicate. The absorbance was converted to the amount of p-nitrophenol released using a p-nitrophenol standard curve and the enzyme activity is determined from the following equation:

(2) $$\begin{array}{c} \alpha -\text{glucosidase activity}=\frac{\text{pNP}\left(mg\right)\times \text{DF}}{T\times E\left(mg\right)}. \end{array}$$

1. pNP (mg) = amount in mg of p-nitrophenol released
2. DF = dilution factor
3. *T* = time of incubation
4. *E* (mg) = amount in mg of enzyme in the reaction mixture

Percentage of enzyme activity was determined by using the following equation:

(3) $$\begin{array}{c} \%\text{ enzyme activity}=\frac{\text{enzyme activity}\left(\text{with AEPAS}\right)}{\text{enzyme activity}\left(\text{without AEPAS}\right)}\times 100. \end{array}$$

A graph of percentage enzyme activity was plotted against the concentrations of AEPAS and the concentration of AEPAS that inhibits 50% enzyme activity (IC50) was determined from the graph.

#### 2.3.6. Determination of IC_50_ of Fractions and Subfractions

**Figure 1:**
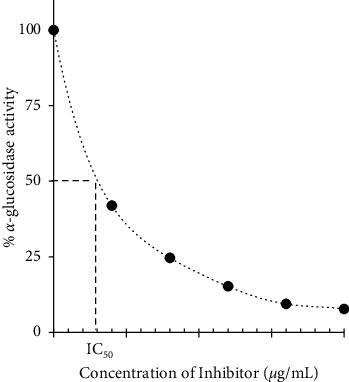
Determination of IC50 from a plot.

The IC_50_ values were determined from the plot of percentage *α*-glucosidase activity against the concentrations of AEPAS, fractions, and subfractions as shown in Figure 1.

#### 2.3.7. Fourier Transform Infrared Spectroscopy (FTIR) Analysis Procedure

FTIR (Cary630) from Agilent Technology, Malaysia, was used for the analysis, and the method of Griffiths and De-Haseth [28] was followed with slight modification. The instrument was powered on and waited until the initialization was completed. The sample holder was cleaned with tissue paper moistened with acetone. The spectrum software on the computer was set up to pop up “Scan and Instrument Setup” dialog. The sample name and the scan range limit (650–4500 cm^−1^) were input.

The “background” icon was clicked to collect the background information of the sample holder before the sample was placed on the sample holder. The “monitor” icon on the “Scan and Instrument Setup” the dialog was activated, and then, the pressure arm was lowered down to the sample until a sound was heard and then “finish” button was activated. “Apply” and then “Start” icons were activated to collect the spectrum. Data of the spectrum obtained were processed by clicking on the “view” and then on the “label peaks” icons to select and label the spectrum with the desired or special peaks. The data were then saved as pdf files.

#### 2.3.8. Gas Chromatography Mass Spectroscopy (GCMS) Analysis Procedure

GC-MS (19091S-433UI) from Agilent Technologies, United States, was used for the analysis. The sample solution was injected into the GC inlet where it was vaporised and swept onto a chromatographic column by the carrier gas, helium. The sample flow through the column and the compounds comprising the mixture of interest were separated by their relative interaction with the coating of the column (stationary phase) and the carrier gas (mobile phase). The latter part of the column passed through a heated transfer line and ended at the entrance to the ion source where compounds eluting from the column were converted to ions.

The ionized compounds were channelled to a filter (mass analyser), where the positively charged ions were separated according to their different mass-related properties, which then entered a detector. The detector then transmitted the information to a computer that recorded all the data produced. The recorded data are then converted electrical impulses into visual display and hard copy display.

#### 2.3.9. Statistical Analysis

The data obtained were analysed using Microsoft Excel 2016 package and reported as values ± standard deviation. Significant levels of the values were compared at *p* > 0.05.

## 3. Results

### 3.1. Activity of Pooled Fractions on *α*-Glucosidase Inhibition

**Figure 2:**
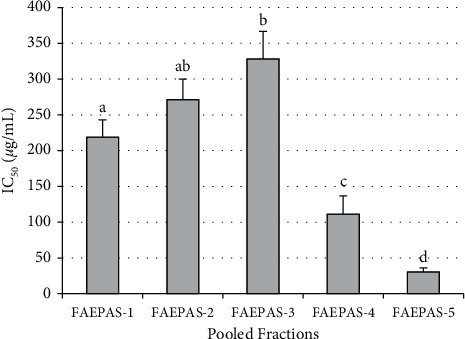
IC_50_ of pooled fractions. NB: columns with different superscript alphabets are significantly (*p* < 0.05) different from each other.

The result of *α*-glucosidase inhibitory activity of the pooled fractions is presented in Figure 2. The result shows that fraction 5 of aqueous extract of *Persea americana* seed (FAEPAS-5) has the lowest IC_50_ against *α*-glucoside, hence the most potent.

### 3.2. *α*-Glucosidase Inhibitory Activity of Subfractions

**Figure 3:**
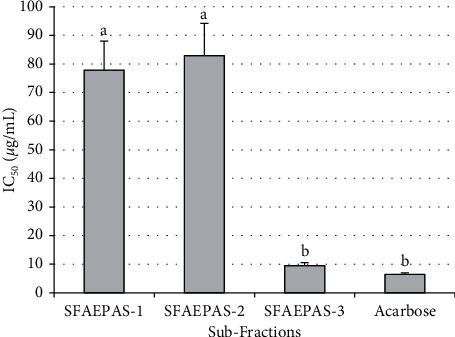
IC_50_ of subfractions. NB: columns with different superscript alphabets are significantly (*p* < 0.05) different from each other.

The results of inhibitory effects and IC_50_ of subfractions of aqueous extract of *Persea americana* seed (SFAEPAS) on *α*-glucosidase activity are presented in Figure 3. Subfraction-3 (SFAEPAS-3) shows the lowest IC_50_, 09.48 ± 1.06, thereby being the most active subfraction. However, acarbose (standard drug) with IC_50_ 06.45 ± 0.47 was slightly more potent than the most active subfraction.

### 3.3. Infrared Spectrum of SFAEPAS-3

**Figure 4:**
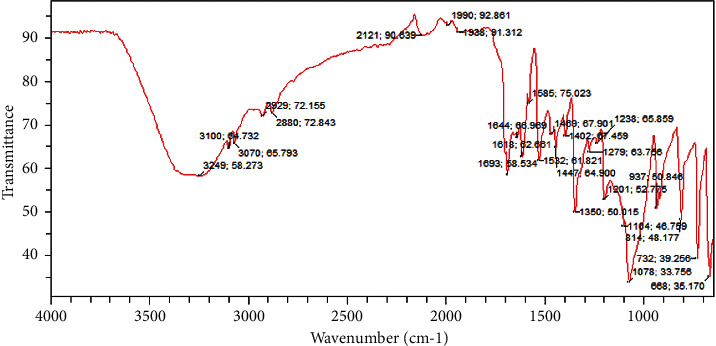
Infrared spectrum of SFAEPAS-3.

The infrared spectrum of the most active subfraction, SFAEPAS-3, from Fourier transform infrared (FTIR) is depicted in Figure 4 and its interpretation is given in Table 1.

### 3.4. GC-MS Chromatogram of SFAEPAS-3

**Figure 5:**
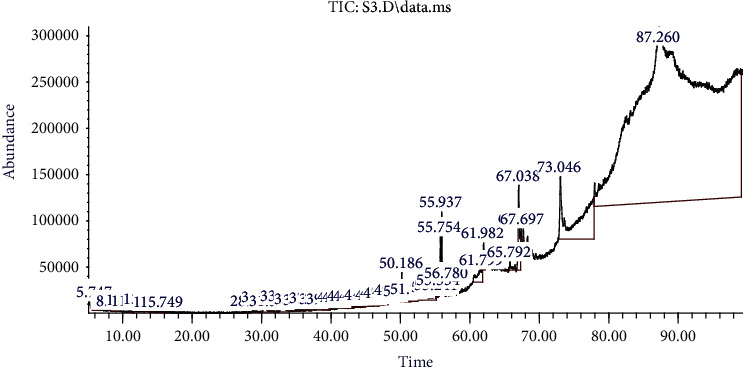
Infrared spectrum of SFAEPAS-3.

Figure 5 depicts the Infrared Spectrum of SFAEPAS-3 from GC-MS analysis and the possible components present are depicted in Table 2, which shows the retention time, percentage area, and suggested names from NIST (2014) library.

Possible components of SFAEPAS-3 from GC-MS analysis showing the retention time, percentage area, and suggested names from NIST (2004) library are presented in Table 2. This shows that cis-11,14-eicosadienoic acid has the highest percentage area (91.03%) and retention time (RT) followed by catechin (4.74%).

**Figure 6:**
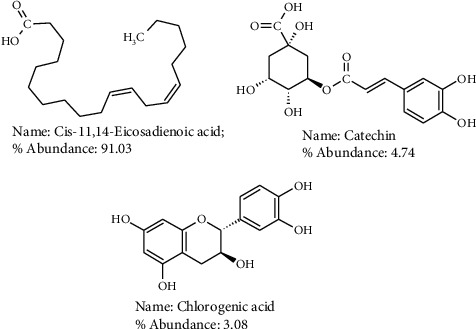
Suspected compounds found in SFAEPAS-3 through GC-MS.

Structures of the suspected compounds in SFAEPAS-3 and their percentage abundance are shown in Figure 6. The compounds are cis-11,14-eicosadienoic acid, catechin, and chlorogenic acid, which were present in 91.03, 4.74, and 3.08 abundantly, respectively.

## 4. Discussion

The practice of herbal medications as complementary approaches to modern-day medications for the treatment of diabetes and its complications is growing globally. One curative tactic for averting diabetes mellitus is to slow down the absorption of glucose through inhibition of *α*-glucosidase, which is the crucial digesting enzyme of carbohydrates. This enzyme is situated in the brush borders of the small intestine. In this study, the inhibitory activity of AEPAS on *α*-glucosidase was revealed. The findings showed that AEPAS possesses significant (*p* < 0.05) inhibitory activity on *α*-glucosidase with IC_50_ close to that of a standard drug (acarbose). IC_50_ is a functional strength of an inhibitor and is not an indicator of its affinity, and it is the concentration required to produce 50% inhibition of an enzyme activity [29]. The lower the IC_50_, the higher the potency of the inhibitor and vice versa. The inhibitory activity exhibited by AEPAS may be due to the nature of the chemical principles or components contained in it.

The most abundant constituents in SFAEPAS-3 which are cis-11,14-eicosadienoic acid (91.03%); catechin (4.74%) and chlorogenic acid (3.08%) have been reported to have hypoglycaemic potentials or inhibitory activities against *α*-glucosidase activity. cis-11,14-Eicosadienoic acid was reported to be one of the major components that inhibits *α*-amylase activity in vitro [30]. This finding is in support of the research conducted by Sukiman et al., [31] which showed that three unsaturated fatty acids—palmitoleic, linoleic, and linolenic acid—were strong inhibitors against *α*-glucosidase. In addition, their reports showed that unsaturated fatty acids have higher inhibitory activities than their counterpart saturated fatty acids and the methyl ester forms of unsaturated fatty acids. That is, the methyl ester forms of unsaturated fatty acids were slightly less active than the free acids [31]. Many other findings are in support of the claims of other scientific researchers that report that long chain unsaturated fatty acids are strong inhibitors of *α*-amylase and *α*-glucosidase [31–36].

Catechin, on the other hand, is a flavan-3-ol, a type of natural phenol and antioxidant. It is a plant secondary metabolite. It belongs to the group of flavan-3-ols (or simply flavanols), part of the chemical family of flavonoids. Catechin possesses two benzene rings (called the A- and B-rings) and a dihydropyran heterocycle with a hydroxyl group on carbon 3. The A ring is similar to a resorcinol moiety, while the B ring is similar to a catechol moiety. A study has shown that dihydroxyl groups at positions C-3′ and C-4′ of flavonoids are effectively conjugated to the active site residues of *α*-glucosidase [37]. Existence of a catechol system on the B ring of flavonoids is expected to contribute to the distribution of the electron cloud, which then becomes accessible to donate hydrogen atoms to form hydrogen bonds with the active site residues of *α*-glucosidase, thereby playing a crucial role in inhibiting its action [37]. Catechin has significant inhibitory activity on both *α*-amylase and *α*-glucosidase [38–40]. Catechin structure resembles that of quercetin that has also been reported to have potent inhibitory activities on *α*-amylase and *α*-glucosidase [39], only that carbon-1 of catechin has no ketone function as in quercetin.

Chlorogenic acid is an ester of caffeic acid and quinic acid, and it is the main polyphenolic compound in coffee [41]. This compound, long known as an antioxidant, has been reported to slow down the release of glucose into the bloodstream after a meal (i.e., postprandial anti-hyperglycaemia) [41]. In vitro reports have shown that coffee phenolics, especially chlorogenic acid, may have similar effects as the drug acarbose in inhibiting *α*-amylase and *α*-glucosidase activities, and that its structure resembles that of acarbose [42, 43]. Chlorogenic acid was reported to be a potent inhibitor of both *α*-amylase and *α*-glucosidase enzymes with IC_50_ of greater than 100 *μ*M [42, 44, 45].

In conclusion, after fractionation, subfraction-3 of *Persia americana* seeds (SFAEPAS-3) showed prominent inhibitory activity against *α*-glucosidase by having the least IC_50_. SFAEPAS-3 was found to be as potent as acarbose, a synthetic medicine, by having almost the same IC_50_. Major components or principles obtained from SFAEPAS-3 through GC-MS were reported to be potent inhibitors of *α*-glucosidase enzyme and hence have great hypoglycaemic potential. This strategy can be used to design novel drugs, which can be used to manage or treat diabetes mellitus. These components can be synthesised in vitro, and their hypoglycaemic potential is determined both in vitro and in vivo.

## Figures and Tables

**Table 1:** Functional groups of compounds present in SFAEPAS-3 using FTIR.

Functional group	Class of compound	Peak (cm^−1^)	Intensity
C=O	Carboxylic acid	1693	Strong, stretched
O–H	Carboxylic acid	3249	Strong, broad
C–O	1º alcohol	1078	Strong, stretched
C=C	Double bonds	1618	Medium, stretched
CH_2_, CH_3_	Long chain fatty acid	29259, 2880	Stretching

**Table 2:** Possible components of SFAEPAS-3 from GC-MS analysis and suggested names from NIST (2004) library.

Area (%)	Library/ID	Ref (NIST, 2014)	RT	CAS	Qual
91.03	cis-11,14-Eicosadienoic acid	180766	87.2602	2463-02-7	92
4.74	Catechin	140258	73.046	154-23-4	83
3.08	Chlorogenic acid	199068	67.697	327-97-9	90

## Data Availability

All data for this study are available on request.

## References

[1] Alhassan A. J., Sule M. S., Et-ta’alu A. B., Lawal A. T. (2017). In vitro inhibitory activities of *Persea americana* seed extracts on *α*-amylase and *α*-glucosidase. *Bayero Journal of Pure and Applied Science*.

[2] Neelesh M., Sanjay J., Sapna M. (2010). Antidiabetic potential of medicinal plants. *Acta Poloniae Pharmaceutica-Drug Research*.

[3] Kumar D. S., Sharathnath V. K., Yogeswaran P. (2010). Medicinal potency of *Momordica charantia*. *International Journal of Pharmaceutical Sciences Review and Research*.

[4] Lawal T. A., Ononamadu C. J., Okonkwo E. K. (2021). In vitro and in vivo hypoglycaemic effect of *Camellia sinensis* on alpha glucosidase activity and glycaemic index of white bread. *Applied Food Research*.

[5] Chinenye S., Young E. (2011). State of diabetes care in Nigeria. *The Nigerian Health Journal*.

[6] Ekam V. S., Ebong P. E., Johnson J. T., Dasofunjo K. (2013). Effect of activity directed fractions of *Vernonia amygdalna* on total body weight and blood glucose levels of diabetic Wistar albino rats. *International Journal of Science and Technology*.

[7] Patel K. P., Joshi H. M., Majmudar F. D., Patel V. J. (2013). Newer Approaches in the treatment of diabetes mellitus. *NHL Journal of Medicinal Science*.

[8] Morrish N. J., Wang S. L., Stevens L. K., Fuller J. H., Keen H. (2001). Mortality and causes of death in the WHO multinational study of vascular disease in diabetes. *Diabetologia*.

[9] Adefegha S. A., Oboh G. (2012). In vitro inhibition activity of polyphenol-rich extracts from *Syzygium aromaticum* (L.) Merr. & Perry (Clove) buds against carbohydrate hydrolyzing enzymes linked to type 2 diabetes and Fe^2+^-induced lipid peroxidation in rat pancreas. *Asian Pacific Journal of Tropical Biomedicine*.

[10] Alhadramy M. S. (2016). Diabetes and oral therapies: a review of oral therapies for diabetes mellitus. *Journal of Taibah University Medical Sciences*.

[11] Alhassan Adamu J., Lawal Tajudeen A., Dangambo M. A. (2017). Antidiabetic properties of thirteen local medicinal plants in Nigeria-a review. *Journal of Pharmaceutical Research*.

[12] Kumar K., Rajput (2018). Current trends of medicinal plants with potential antidiabetic activity: a review. *Paripex-Indian Journal of Research*.

[13] Terra W. R., Ferreira C. (2005). Biochemistry of digestion. *Comprehensive Molecular Insect Science*.

[14] Abbas G., Al-Harrasi A. S., Hussain H. (2017). *α*-glucosidase enzyme inhibitors from natural products. *Discovery and Development of Antidiabetic Agents from Natural Products*.

[15] Piotr T., Horton D. (2012). Enzymatic conversions of starch. *Advances in Carbohydrate Chemistry and Biochemistry*.

[16] Bedekar A., Shah K., Koffas M. (2010). Natural products for type II diabetes treatment. *Advances in Applied Microbiology*.

[17] Brai B., Adisa R. A., Odetola A. A. (2014). Hepatoprotective properties of aqueous leaf extract of *Persea americana* mill (Lauraceae) “avocado” against CCl_4_-induced damage rats. *African Journal of Traditional, Complementary and Alternative Medicines*.

[18] Nwauzoma A. B., Dappa M. S. (2013). Ethnobotanical studies of port Harcourt metropolis, Nigeria. *International Scholarly Research Notices*.

[19] Yasir M., Das S., Kharya M. D. (2010). The Phytochemical and pharmacological profile of *Persea americana* mill. *Pharmacognosy Reviews*.

[20] Isaac A. T., Ganiyu O., Akinyemi A. J., Ajani R. A., Olanrewaju B. O. (2014). Avocado pear fruit and leaves aqueous extracts inhibit *α*-amylase, *α*-glucosidase and SNP induced lipid peroxidation-an insight into mechanisms involve in management of type 2 diabetes. *International Journal of Applied and Natural science (IJANS)*.

[21] Alhassan A. J., Sule M. S., Sule M. S., Atiku M. K., Wudil A. M., Abubakar H. (2012). Effects of aqueous avocado pear (*Persea americana*) seed extract on alloxan induced diabetes rats. *Greener Journal of Medical Sciences*.

[22] Kosińska A., Karamać M., Estrella I., Hernández T., Bartolomé B., Dykes G. A. (2012). Phenolic compound profiles and antioxidant capacity of *Persea americana* Mill. peels and seeds of two varieties. *Journal of Agricultural and Food Chemistry*.

[23] Abdel-Aty A. M., Elsayed A. M., Salah H. A., Bassuiny R. I., Mohamed S. A. (2021). Egyptian chia seeds (*Salvia hispanica* L.) during germination: upgrading of phenolic profile, antioxidant, antibacterial properties and relevant enzymes activities. *Food Science and Biotechnology*.

[24] Abdel-Aty A. M., Bassuiny R. I., Barakat A. Z., Mohamed S. A. (2019). Upgrading the phenolic content, antioxidant and antimicrobial activities of garden cress seeds using solid-state fermentation by *Trichoderma reesei*. *Journal of Applied Microbiology*.

[25] Barakat A. Z., Bassuiny R. I., Abdel-Aty A. M., Mohamed S. A. (2020). Diabetic complications and oxidative stress: the role of phenolic-rich extracts of *Saw palmetto* and date palm seeds. *Journal of Food Biochemistry*.

[26] Mosihuzzman M., Naheed S., Hareem S. (2013). Studies on alpha glucosidase inhibition and antiglycation potential of Iris loczyi and Iris unguicularis. *Life Science*.

[27] Hong H. C., Li S. L., Zhang X. Q., Ye W. C., Zhang Zhang (2021). Flavonoids with alpha glucosidase inhibitory activities and their contents in the leaves of Morus atropurpurea. *Chinese Medicine*.

[28] Griffiths P., De-Haseth J. (1986). *Fourier Transform Infrared Spectrometry*.

[29] Yung-Chi C., Prusoff W. H. (1973). Relationship between the inhibition constant (Ki) and the concentration of inhibitor which causes 50 percent inhibition (IC_50_) of an enzymatic reaction. *Biochemical Pharmacology*.

[30] Conforti F., Menichini F., Loizzo M. R. (2008). Antioxidant, *α*-amylase inhibitory and brine-shrimp toxicity studies on *Centaurea centaurium* L. methanolic root extract. *Natural Product Research*.

[31] Sukiman H., Kardono L. B. S., Tachibana  S., Artanti N. (2012). Isolation of *α*-glucosidase inhibitors produced by an endophytic fungus, Colletotrichum SP. TS13 from Taxus Sumatrana, Pakistan. *Journal of Biological Sciences*.

[32] Artanti N., Tachibana S., Kardono L. B. S. (2014). Effect of media composition on *α*-glucosidase inhibitory activity, growth and fatty acid content in mycelium extracts of Colletotrichum SP. TSC13 from *Taxum sumatrana* (Miq) de Laub. *Pakistan Journal of Biological Sciences*.

[33] Hamden K., Keskes H., Belhaj S., Mnafgui K., Feki A., Allouche N. (2011). Inhibitory potential of omega-3 fatty and fenugreek essential oil on key enzymes of carbohydrate-digestion and hypertension in diabetes rats. *Lipids in Health and Disease*.

[34] Lean-Teik N., Su C., Hsu C. (2013). Inhibitory potential of fatty acids on key enzymes related to type 2 diabetes. *IUBM Journals*.

[35] Nguyen T. H., Kim S. M., Um A. B. (2011). Two unsaturated fatty acids with potent *α*-glucosidase inhibitory activity purified from the body wall of sea cucumber (*Stichopus japonicus*). *Journal of Food Science*.

[36] Nguyen T. H., Kim S. M. (2015). Glucosidase inhibitory activities of fatty acids purified from the internal organ of sea cucumber *Stichopus japonicas*. *Journal of Food Science*.

[37] Sarian M. N., Ahmed Q. U., Siti Z. M. S. (2017). Antioxidant and antidiabetic effects of flavonoids: a structure-activity relationship based study. *BioMed Research International*.

[38] Geng S., Shan S., Ma H., Liu B. (2016). Antioxidant activity and *α*-glucosidase inhibitory activities of the polycondensate of catechin with glyoxylic acid. *PLoS ONE*.

[39] Jang H. K., Hyo Y. K., Seo Y. Y., Jin-Baek K., Chang H. J., Young H. K. (2018). Inhibitory activity of (−)-epicatechin-3,5-O-digallate on *α*-glucosidase and in silico analysis. *International Journal of Biological Macromolecules*.

[40] Meltem Y., Anneke M. G., Alexander J. M., Erik S., Balz F. (2012). Inhibition of *α*-amylase and *α*-glucosidase activity by tea and grape seed extracts and their constituent catechins. *Journal of Agricultural and Food Chemistry*.

[41] Kang J., Liu Y., Xie M. X., Li S., Jiang M., Wang Y. D. (2004). Interactions of human serum albumin with chlorogenic acid and ferulic acid. *Biochimica et Biophysica Acta*.

[42] Karim Z., Holmes M., Orfila C. (2017). Inhibitory effect of chlorogenic acid on digestion of potato starch. *Food Chemistry*.

[43] Tan M. (1997). Alpha-glucosidase inhibitors in the treatment of diabetes. *Currurrent Opinion Endocrinological Diabetes*.

[44] Narita Y., Inouye K. (2009). Kinetic analysis and mechanism on the inhibition of chlorogenic acid and its components against porcine pancreas *α*-amylase isozymes I and II. *Journal of Agricultural and Food Chemistry*.

[45] Oboh G., Agunloye O. M., Adefegha S. A., Akinyemi A. J., Ademiluyi A. O. (2015). Caffeic and chlorogenic acids inhibit key enzymes linked to type 2 diabetes (in vitro): a comparative study. *Journal of Basic and Clinical Physiology and Pharmacology*.

